# The Intestinal Peptide Transporter PEPT1 Is Involved in Food Intake Regulation in Mice Fed a High-Protein Diet

**DOI:** 10.1371/journal.pone.0026407

**Published:** 2011-10-21

**Authors:** Anna-Maria Nässl, Isabel Rubio-Aliaga, Manuela Sailer, Hannelore Daniel

**Affiliations:** ZIEL Research Center of Nutrition and Food Sciences, Abteilung Biochemie, Technische Universität München, Freising, Germany; New Mexico State University, United States of America

## Abstract

High-protein diets are effective in achieving weight loss which is mainly explained by increased satiety and thermogenic effects. Recent studies suggest that the effects of protein-rich diets on satiety could be mediated by amino acids like leucine or arginine. Although high-protein diets require increased intestinal amino acid absorption, amino acid and peptide absorption has not yet been considered to contribute to satiety effects. We here demonstrate a novel finding that links intestinal peptide transport processes to food intake, but only when a protein-rich diet is provided. When mice lacking the intestinal peptide transporter PEPT1 were fed diets containing 8 or 21 energy% of protein, no differences in food intake and weight gain were observed. However, upon feeding a high-protein (45 energy%) diet, *Pept1^−/−^* mice reduced food intake much more pronounced than control animals. Although there was a regain in food consumption after a few days, no weight gain was observed which was associated with a reduced intestinal energy assimilation and increased fecal energy losses. *Pept1^−/−^* mice on high-protein diet displayed markedly reduced plasma leptin levels during the period of very low food intake, suggesting a failure of leptin signaling to increase energy intake. This together with an almost two-fold elevated plasma arginine level in *Pept1^−/−^* but not wildtype mice, suggests that a cross-talk of arginine with leptin signaling in brain, as described previously, could cause these striking effects on food intake.

## Introduction

Numerous studies have demonstrated that diets with a high protein content provide higher satiety levels (at least short term) than the other macronutrients [1], [2]. In addition, it has been shown that protein-rich diets can promote weight loss and cause changes in body composition [1]. Intake of dietary protein is sensed in the intestine with concomitant secretion of gastrointestinal hormones and activation of visceral processes that alter gastric motility, stimulate pancreatic secretion, mediate peripheral effects and contribute to satiety [3]–[5]. Mainly PYY and CCK but also insulin and leptin are discussed to play a prominent role in satiety control [6]–[8]. Yet, in most cases feeding high-protein diets revealed negligible effects on circulating levels of these hormones [9]–[11]. Recently, neuronal pathways in the brainstem nucleus of the solitary tract and hypothalamic arcuate nucleus were shown to be activated by high-protein diets [12]. Amongst the possible dietary signals for this hypothalamic sensing, the amino acid leucine has received particular attention. It was demonstrated that leucine contributes to food intake control via AMP-activated protein kinase and mammalian target of rapamycin when supplied to hypothalamic centers [13], [14]. Since plasma levels of branched chain amino acids (BCAA), including leucine, increase when dietary protein supply is increased and brain leucine levels follow plasma levels, a role of leucine in central regulation of satiety seems plausible. However conflicting results on the role of leucine were obtained in animal studies in which extra leucine was supplied by the diet to affect food intake and body weight [15], [16]. Additionally high-protein intake causes also major adaptations in metabolic processes in intestine and liver associated with changes in plasma levels of a variety of amino acids [17]. Although protein-rich diets challenge the digestive tract with large quantities of amino acids and short chain peptides for uptake into epithelial cells and into circulation, a contribution of intestinal transport processes to food intake control has never been anticipated.

Intestinal protein digestion delivers short chain peptides and free amino acids to epithelial cells. Amino acids are taken up through numerous amino acid transporters acting as symporters or antiporters [18]. For absorption of di- and tripeptides only one transport system in the intestine, designated as PEPT1 (SLC15A1) is known [19]. PEPT1 is a low-affinity but high-capacity transport system and handles essentially all possible protein-derived di- and tripeptides, but also a variety of peptidomimetics like aminocephalosporins and various prodrugs [20]. Peptide transport is electrogenic by charge movement as it involves the cotransport of protons [21]. PEPT1 in the intestine is subject to regulation by a variety of hormones and cytokines [22], but also by the dietary protein content. As demonstrated by Erickson *et al*, mRNA expression and transport rate of PEPT1 in rat intestine increases 1.5 to 2-fold when animals received a high-protein (50 energy%) diet as compared to a 4% protein diet [23].

Although the structure and function of PEPT1 has been studied in detail [21], its contribution to overall amino acid absorption is still unknown. The PEPT1-deficient model organism *Caenorhabditis elegans* showed reduced body size, impaired brood size and a retarded postembryonic development [24]. We recently reported that the lack of intestinal peptide transport in *Pept1^−/−^* mice is not compensated by changes in mRNA expression or transport capacity of intestinal amino acid transporters. Phenotyping of *Pept1^−/−^* mice did not reveal any impairments in reproduction, body weight or any other anthropometric or clinical chemistry measures when animals were fed a standard high-carbohydrate diet [25]. Yet, plasma concentrations of amino acids were increased in *Pept1^−/−^* when compared to *Pept1^+/+^* mice, suggesting an altered systemic amino acid handling. Moreover, administration of an acute high protein load via gastric gavage also caused differences in plasma concentrations of several amino acids such as citrulline and arginine and most prominently of proline [26], [27]. Based on this finding, feeding trials with *Pept1^−/−^* animals with variations in dietary protein content were performed and we here report striking diet-dependent effects on food intake and weight gain that were further characterized by biochemical analysis. Taken together our findings suggest that the intestinal peptide transporter PEPT1 is part of a metabolic network that affects food intake particularly when high-protein diets are consumed.

## Results

### Effect of a high-protein diet on food intake and body weight in *Pept1^−/−^* and *Pept1^+/+^* mice

**Figure 1:**
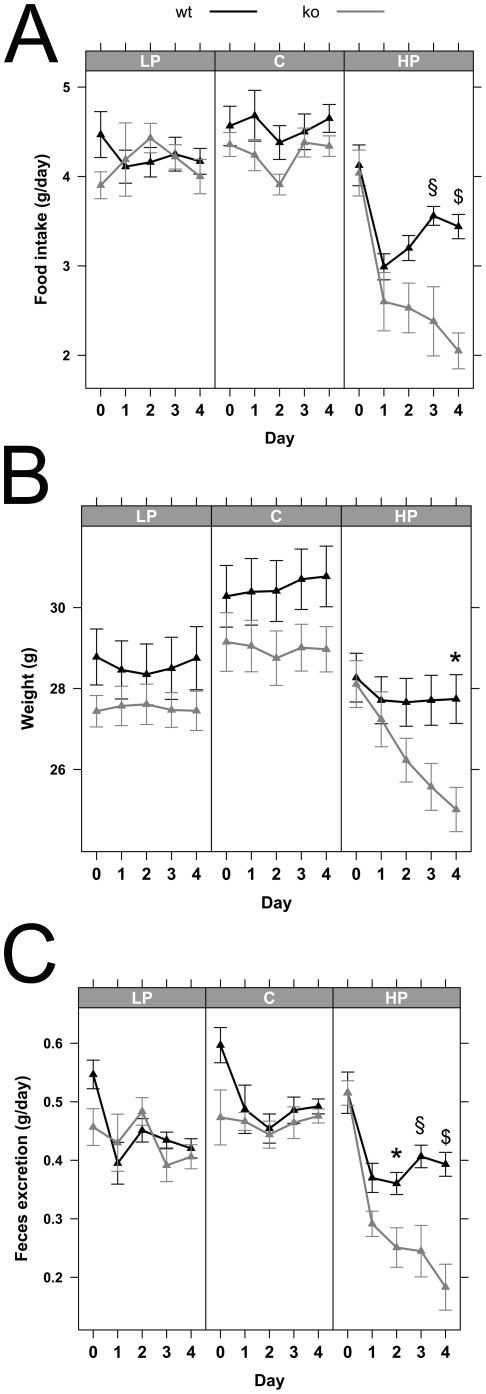
*Pept1^−/−^* animals on high-protein diet show major changes in metabolic parameters. Metabolic parameters of male mice on low-protein (LP  = 8% protein energy), control (C  = 21% protein energy) and high-protein (HP = 45% protein energy) diet were determined in *Pept1^+/+^* (wt) and *Pept1^−/−^* (ko) animals. During 5 days on the different diets, food intake (A), weight (B), and feces excretion (C) were determined (n = 10). Data are presented as mean±SEM. *P<0.05, ^$^P<0.01, ^§^P<0.001.

**Figure 2:**
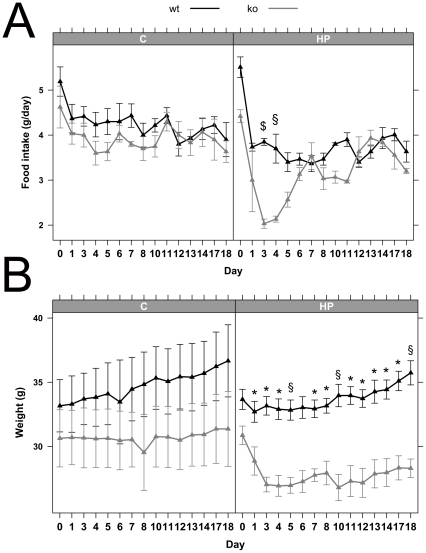
Food intake and body weight during feeding *Pept1^−/−^* animals a high-protein diet. Food intake (A) and body weight (B) of male *Pept1^+/+^* (wt) and *Pept1^−/−^* (ko) mice on control (C = 21% protein energy) or high-protein (HP = 45% protein energy) diet recorded over the 18 days feeding trial. Data are presented as the mean±SEM. *P<0.05, ^$^P<0.01, ^§^P<0.001.

Determination of food intake and body weight changes in *Pept1^−/−^* mice fed for 5 days a LP or C diet did not show any significant alterations when compared to wildtype animals. However when animals were provided with a HP diet, food intake rates immediately declined in all animals but more pronounced in *Pept1^−/−^* animals (Figure 1). Whereas wildtype animals increased food consumption again after 2 days, *Pept1^−/−^* mice reduced food intake even further over 4 days (Figure 1A). This led in *Pept1^−/−^* animals also to a decrease in body weight (Figure 1B) and reduced feces excretion (Figure 1C). No differences in water consumption were observed, neither between diets nor genotypes (data not shown). A second feeding trial conducted over a 18 day period revealed a similar initial major reduction in food intake for 4 days in *Pept1^−/−^* mice while animals thereafter increased food consumption to reach the same intake rates as observed in wildtype animals (Figure 2A), yet, they failed to show any significant weight gain over the entire feeding trial (Figure 2B).

Analysis of energy content in feces determined by bomb calorimetry revealed significantly higher energy losses in *Pept1^−/−^* than in *Pept1^+/+^* animals. At day 5 on the HP diet energy density in feces accounted to 12.20 kJ/g in wildtype animals and 15.23 kJ/g (*P*<0.001) in *Pept1^−/−^* mice and these differences remained to the end of the experiment accounting at day 18 to 12.19 kJ/g feces in *Pept1^+/+^* and 14.68 kJ/g feces (*P*<0.001) in *Pept1^−/−^* mice. In addition, energy balance calculations with the data from the 5 day feeding trial revealed that reduced energy assimilation and increased fecal caloric losses cause a difference of 71 kJ less energy available to PEPT1-deficient animals on the HP diet (Table 1).

**Table 1:** Energy assimilation in *Pept1^+/+^* and *Pept1^−/−^* animals on low-protein (LP = 8% energy from protein), control (C = 21% energy from protein) or high-protein (HP = 45% energy from protein) diet.

Group	Genotype	Sum of energy intake(kJ)	Sum of energy excretion (kJ)	Sum of energy assimilation (kJ)	Energy difference over 5 days (kJ)
LP	*Pept1^+/+^*	387.23	27	360.23	7.62
	*Pept1^−/−^*	379.6	26.66	352.61	
C	*Pept1^+/+^*	409.98	29.83	380.15	28.36
	*Pept1^−/−^*	382.08	30.29	351.79	
HP	*Pept1^+/+^*	351.53	25.16	326.37	71.05
	*Pept1^−/−^*	276.08	20.76	255.32	

The given values are the sum of the 5 day lasting feeding trial. Energy calculations of *Pept1^+/+^* and *Pept1^−/−^* animals on low-protein (8% protein energy), control (21% protein energy) and high-protein (45% protein energy) diet for 5 days were analyzed (n = 10). Data are presented as sum of energy intake, energy excretion and energy assimilation over 5 days of feeding the diets.

### Changes in plasma parameters in *Pept1^−/−^* mice

**Figure 3:**
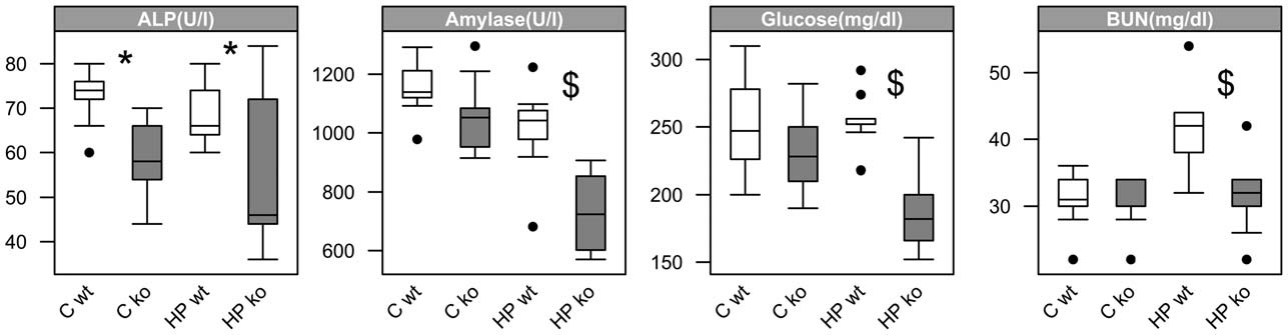
Decreased activities of enzymes and glucose in plasma of *Pept1^−/−^* animals. Enzyme and metabolite activities in plasma of *Pept1^+/+^* (wt) and *Pept1^−/−^* (ko) animals fed a control (C = 21% protein energy) or high-protein (HP = 45% protein energy) diet were analyzed. Alkaline phosphatase (ALP), amylase, glucose and blood urea nitrogen (BUN) were determined after 5 days on the high-protein diet (n = 10). Data are presented as mean±SD. (•) indicate outlier. *P<0.05, ^$^P<0.01, ^§^P<0.001.

Clinical chemistry data of mouse plasma collected after 5 days revealed lower alkaline phosphatase levels in *Pept1^−/−^* animals, both on C and HP diet. In addition *Pept1^−/−^* mice, when fed the HP diet displayed also significantly decreased levels of amylase and glucose. In contrast, wildtype animals on HP diet showed higher blood urea nitrogen levels when compared to mice on C diet (HP: 41.8±6.2 mg/dl vs. C: 31±4.1 mg/dl, P<0.001), whereas blood urea nitrogen levels in *Pept1^−/−^* animals on HP diet were comparable to those on C diet (HP: 31.8±5.6 mg/dl vs. C: 30.4±3.8 mg/dl; *P*<0.001). These data are summarized in Figure 3. When blood urea nitrogen levels were determined after 18 days, genotype effects were no longer detectable and enyzme activities did also not show any more differences (data not shown). Yet, independent of genotype, mice on the HP diet displayed significant elevated blood urea nitrogen concentrations (HP: 44±4.3 mg/dl; vs. C: 26±3.6, mg/dl; *P*<0.001).

### High-protein diet alters plasma levels of amino acids and derivatives

**Figure 4:**
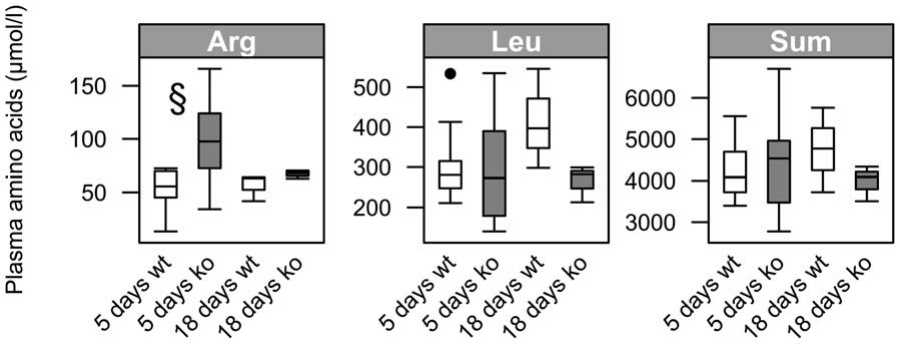
Changed plasma amino acids after 5 and 18 days on high-protein diet in *Pept1^−/−^* animals. By LC-MS/MS plasma amino acid concentrations of *Pept1^+/+^* (wt) and *Pept1^−/−^* (ko) animals a high-protein (45% protein energy) diet were analyzed (n = 3–10). Plasma amino acid profiles of arginine (Arg), leucine (Leu) and the sum of all detectable amino acids (Sum) of *Pept1^−/−^* (ko) and *Pept1^+/+^* (wt) animals on high-protein (HP) diet for 5 and 18 days are depicted. Data are presented as mean±SD. (•) indicate outlier. *P<0.05, ^$^P<0.01, ^§^P<0.001.

To assess alterations in systemic amino acid levels of mice fed diets with different protein contents, we analyzed plasma samples by LC-MS/MS. As a highly consistent and genotype-specific finding, we observed an almost two-fold increase in plasma levels of arginine in *Pept1^−/−^* mice on both, C (ko: 90±29.8 µmol/l vs. wt: 44.8±22.8 µmol/l, *P* = 0.002) and HP diet (ko: 96.9±42.6 µmol/l vs. wt: 53.1±19.1 µmol/l, *P* = 0.008) after 5 days (Table S1). Genotype-independent effects of the diets were found for valine, leucine, isoleucine, but also for alpha-aminobutyric acid and glutamine with significantly increased levels in plasma obtained from animals on the HP diet. Decreased plasma levels were found for anserine and hydroxyproline. After 18 days of feeding, alpha-aminobutyric acid levels as well as leucine and isoleucine levels remained increased (Table S2) whereas glutamine and ethanolamine showed higher levels in animals on control than on HP diet irrespective of genotypes. Representative plasma amino acid levels at days 5 and 18 when feeding the HP diet are summarized in Figure 4.

### Altered hepatic amino acid levels and enzyme activities in *Pept1^−/−^* animals

Amino acid levels and enzyme acitivities in liver tissue samples were determined and revealed that after 18 days of feeding the HP diet, 10 out of 28 quantified metabolites were changed (Table S3) with significantly decreased concentrations of methionine, citrulline and taurine but increased levels of leucine, tyrosine, serine, asparagine, ethanolamine, gamma-aminobutyric acid and the urea cycle intermediate ornithine. However, as shown in Table 2, neither urea levels in liver nor hepatic enzyme activities revealed any genotype- or diet-specific effects.

**Table 2:** Enzyme activities and urea levels in liver tissue of *Pept1^+/+^* and *Pept1^−/−^* animals after feeding a high-protein diet (45% energy from protein) for 18 days.

Parameter	Control		High-protein	
	*Pept1^+/+^*	*Pept1^−/−^*	*Pept1^+/+^*	*Pept1^−/−^*
**GDH (mU/mg)**	752.3±109.8	738.0±79.8	708.7±82.7	955.8±119.2
**AST (mU/mg)**	2422.7±338.2	2475.1±560.2	1965.8±189.9	2141.8±351.2
**ALT (mU/mg)**	784.3±45.7	797.2±18.3	623.4±19.4	643.2±65.0
**Urea (µmol/mg)**	13.9±0.9	10.0±0.9	16.1±2.1	15.0±4.0

Enzyme activities of glutmate dehydrogenase (GDH), alanine aminotransferase (AST), alkaline aminotransferase (ALT) as well as urea levels were determined in liver tissue collected from *Pept1^+/+^* and *Pept1^−/−^* animals kept on standard diet (21% protein energy) and high-protein (45% protein energy) diet. All data are presented as mean ± SD (n = 3 animals). P-value was obtained by unpaired Student's t-test.

### Concentrations of ^14^N and ^15^N-lableled amino acids in portal blood after ^15^N-protein gavage

To determine whether any changes in intestinal amino acid absorption and/or metabolism can be detected, portal vein plasma of *Pept1^+/+^* and *Pept1^−/−^* mice was analyzed by LC-MS/MS. Plasma samples were obtained 15 and 30 min after administration of a ^15^N-labeled yeast protein extract provided by gavage. For ^14^N-labeled amino acids differences between genotypes were only detectable for lysine after 15 min (ko: 294.8±22.1 µmol/l vs. wt: 197.6±42.2 µmol/l, *P* = 0.002) and arginine after 30 min (ko: 36.6±4.1 µmol/l vs. wt: 25.1±7.6 µmol/l, *P* = 0.02). For ^15^N-labeled amino acids in portal blood only isoleucine showed slightly reduced concentrations with a genotype-specific effect at 15 min (ko: 29.8±4.2 µmol/l vs. wt: 36.6±3.8 µmol/l, *P* = 0.03) but none of the other amino acids. Taken together, neither levels of ^14^N nor ^15^N-labeled amino acids determined in portal blood of *Pept1^+/+^* and *Pept1^−/−^* mice revealed remarkable differences (Figure S1) that could have indicated altered amino acid delivery to circulation.

### Decreased leptin levels in *Pept1^−/−^* animals

**Figure 5:**
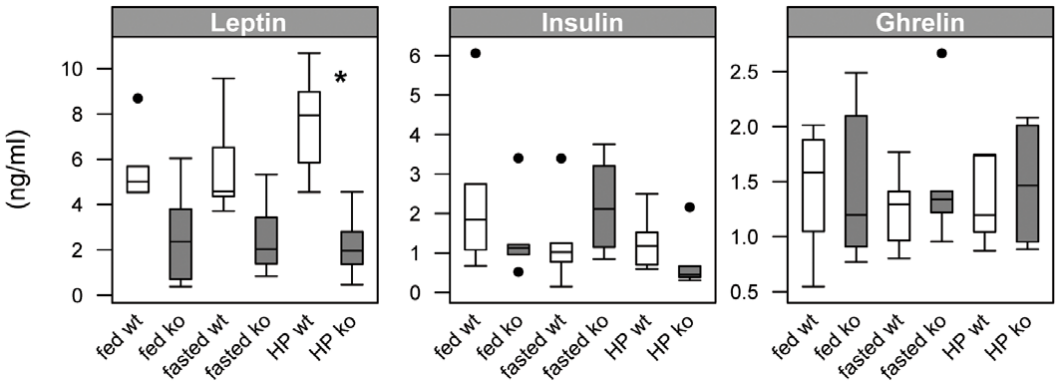
Analysis of plasma hormone levels reveals lower leptin concentrations in *Pept1^−/−^* animals. Plasma samples of fasted (6 h fasting period) and non-fasted *Pept1^+/+^* (wt) and *Pept1^−/−^* (ko) animals fed a control (C = 21% protein energy) diet or a high-protein (HP = 45% protein energy) diet were analysed after 24 h for changes in ghrelin, leptin and insulin levels (n = 6 animals). Data are presented as mean±SD. (•) indicate outliers. *P<0.05, ^$^P<0.01, ^§^P<0.001.

To assess whether selected hormones or adipokines revealed changes that could be associated with the differences in food intake, ghrelin, insulin and leptin levels were determined. As shown in Figure 5, neither ghrelin nor insulin levels changed, whereas leptin levels differed significantly between *Pept1^+/+^* and *Pept1^−/−^* mice. Plasma leptin levels dropped significantly in PEPT1-deficient animals on the HP diet after 24 h to 2.2±1.4 ng/ml as compared to wildtype animals with 7.6±2.4 ng/ml (*P* = 0.001). This effect, yet not reaching significance was also observed on control diet in both, the fed (ko: 2.6± 2.1 ng/ml vs. wt: 5.6±1.6 ng/ml) and the fasted state (ko: 2.5±1.7 ng/ml vs. wt: 5.7±2.4 ng/ml).

## Discussion

Energy intake is strongly affected by diet composition and numerous studies in animals and humans have demonstrated that protein-rich diets initiate higher satiety levels than other macronutrients [1], [28]–[31]. Amongst the various mechanisms proposed to mediate this effect (for review see Thomé [32]) two hypotheses have recently received considerable attention. One involves the hypothalamic sensing of the increased leucine availability on high-protein diets, the other is based on an increased intestinal gluconeogenesis sensed in the portal system and transmitted via afferences of the vagus nerve to brain. We here provide evidence that intestinal transport mechanisms for nutrients involving the intestinal peptide transporter PEPT1 participate in the control of food intake on a high-protein diet. Only in response to a high-protein (45 energy%) diet, *Pept1^−/−^* animals showed a much more pronounced reduction in food intake than wildtype animals. Although *Pept1^−/−^* mice increased food intake again after 5 days on the HP diet with a similar daily caloric intake as wildtype animals, body weight reduction in *Pept1^−/−^* animals sustained, whereas wildtype animals increased body weight after a few days. In parallel to the decline in food intake, body weight decreased with a regain after 4 to 5 days when food intake increased again. Despite very similar food and thus caloric intake after the regain, a significantly increased energy loss in feces on all diets and in particular on the HP diet in *Pept1^−/−^* mice was observed. This suggests that energy assimilation in the intestine is modestly impaired and that the lack in weight gain on the high-protein diet, despite similar food intake rates as in control animals after 5 to 6 days is a consequence of this. However, the data also suggest that *Pept1^−/−^* mice are unable to regulate energy intake adequately for preventing weight loss or for increasing body weight after the regain of food intake. To assess whether hormones that a part of satiety sensing are altered in PEPT1-deficient mice we analyzed non-fasting and fasting plasma ghrelin and insulin levels but did not observe any differences, neither between diets nor genotypes. In contrast, leptin, a key signaling molecule in energy homeostasis and regulation of food intake [33] displayed significantly decreased plasma levels in *Pept1^−/−^* animals on the high-protein diet. Leptin levels generally follow fat mass changes but also decline quickly following food deprivation. Our data suggest that a PEPT1-deficiency could antagonize leptin actions as low plasma leptin levels should lead to an increase in food intake. Most interestingly, leptin was shown to regulate PEPT1 expression and to alter its transport capacity in the intestine [34]–[36] arguing that amino acid absorption in the intestine mediated by PEPT1 could be part of the body's response to changes in leptin levels that participate in control of energy homeostasis.

Various studies have proposed that signalling processes initiated in the portal vein and transmitted from there via afferences of the vagus nerve to brain may provide satiety signals [12], [32], [37]. Such a mechanims was also suggested for the hypophagic effects of high-protein diets via an increased intestinal gluconeogenesis rate and elevated portal glucose concentrations in rats [38]–[40]. However, these findings have been challenged by a lack of evidence for a significant intestinal gluconeogenesis from the stable-isotope labeled glucogenic amino acid glutamine in two strains of fasted rats [41]. This confirmed other studies, that could also not demonstrate a significant intestinal glucose production in rats fed a high-protein diet [42]. We recently reported that there were essentially no differences in the apperance rates of amino acids in the portal vein and peripheral plasma between wildtype and PEPT1-deficient animals [26]. We also administered a ^15^N-labeled yeast extract to wildtype and PEPT1-deficient animals by gavage and here report the extended analysis of samples for stable isotope labeled amino acids in portal blood with emphasis on glucogenic and non-proteinogenic amino acids. Since there were no detectable differences between genotypes in any of the relevant amino acids we conclude, that despite the questionable role of intestinal gluconeogenesis, this process seems not to account to the satiety effect of the high-protein diet in PEPT1-deficient animals reported here.

In addition to peptide uptake, mediated by PEPT1, a large number of apical and basolateral amino acid transporters contribute to amino acid delivery into systemic circulation (for reviews see [18], [43]). After portal delivery, selective hepatic extraction of amino acids allows only some of the 20 proteinogenic amino acids to change in peripheral blood in the absorptive phase. Amongst them are the BCAA as well as the aromatic amino acids. Especially the BCAA leucine has been proposed to contribute to satiety control. Administration of leucine into hypothalamic regions in rats was shown to increase brain mTOR signaling leading to a decrease in food intake and body weight suggesting that low leucine levels in brain may initiate mechanisms that stimulate food intake whereas high levels can increase satiety [13]. However, this finding contradicts with clinical experiences in various human diseases in which supplementing BCAA in patients generally caused an increase in food intake [44], [45]. Moreover, a supplementation of leucine in a diet with otherwise normal protein content failed to show in mice any effect on food intake or weight gain [46]. When provided in drinking water to mice fed either a control or a high-fat diet, leucine supplementation did also not reveal effects on food intake but improved some metabolic parameters in animals on the high-fat diet [47]. In the present study plasma amino acid profiling in animals on high-protein or control diet after 5 or 18 days revealed that plasma BCAA and in particular leucine levels increased significantly on the high-protein diet (P = 0.02) without differences by genotype. This means that despite almost identical plasma leucine levels, *Pept1^−/−^* mice reduced food intake much more pronounced than wildtype animals over the first 5 days of feeding the HP diet, suggesting that increased plasma leucine per se cannot account for the effects of HP diets on satiation.

To assess whether any changes in liver metabolism can be associated with the diet effects in PEPT1-deficient animals we analyzed hepatic amino acid levels and enzyme activities. Protein-rich diets require increased nitrogen elimination from amino acid oxidation which is achieved by an increased delivery of nitrogen to liver via glutamine, glutamate and alanine, an enhanced urea cycle flux and increased renal excretion of urea. Blood urea concentrations increased as expected in animals fed the HP diet but were significantly lower in *Pept1^−/−^* animals (Fig. 3), while liver urea levels did not reveal any genotype effect despite the diet-effects. A recent proteome analysis of hepatic proteins in mice fed a normal or a high-protein diet [48] identified carbamoylphosphate synthetase 1 and ornithine aminotransferase with increased protein levels, suggesting that the increased ornithine demand is achieved from proline while ornithine-transcarbamoylase-activity may limit efficient urea cycle flux. We here observed that citrulline levels in liver tissue declined while ornithine levels increased upon high-protein feeding, but without a genotype-specific effect. Although liver arginine was below detection limit, *Pept1^−/−^* mice displayed significantly increased plasma arginine levels on both, control as well as high-protein diet at days 5 and 18. Plasma citrulline levels were also significantly higher in *Pept1^−/−^* animals at day 5 but no longer at day 18. Although portal blood arginine and citrulline levels did not reveal any genotype-specific differences, arginine concentrations in portal blood were much lower than those in peripheral blood. This suggests that the unusual high plasma levels of arginine in *Pept1^−/−^* mice originate most likely from an increased renal arginine production from citrulline. In this respect it is of interest that dietary L-arginine supplementation (but not D-arginine) was shown to reduce food intake in mice and this was attributed to altered nitric oxide (NO) levels in brain [49]. Since brain arginine concentrations were shown to change almost proportional to plasma arginine levels [50], it is suggested that *Pept1^−/−^* mice may have also an increased arginine level in brain. Most interestingly, delivery of arginine to brain was shown to antagonize the leptin effects on food intake in mice with a prominent regain of food consumption when co-administered centrally with leptin [51]. Moreover, this effect was abolished in NO-Synthase (nNOS) deficient mice and nNOS is shown to be upregulated by leptin [52] and down-regulated by food deprivation [53]. A similar effect of leptin on brain NO production and food intake has also been shown in chicken [54], [55]. Since we observed low leptin levels in *Pept1^−/−^* mice only when animals received the HP diet and when food intake was markedly reduced while animals had almost two-fold higher plasma arginine levels than wildtype controls, it is tempting to speculate that the leptin-NOS axis in brain contributes to the changes in food intake on protein-rich diets. Low leptin levels in PEPT1-deficient mice on the HP diet most likely arise from the very low food intake and the apparent ‘starvation condition’. Plasma leptin levels are known to decrease radiply upon starvation, yet, low leptin levels should increase food intake and this response could be antagonized by the high plasma arginine levels in *Pept1^−/−^* mice. Assuming in analogy to previous studies that the low leptin levels in *Pept1^−/−^* mice and the food deprivation cause major changes in NOS levels [51], [53], then NO production could in spite of the high arginine levels, be reduced. This would be in line with the reported anorexic effects of inhibitors of NOS [56], [57]. Although we cannot provide conclusive evidence for this hypothesis, our findings call for future studies employing agonist and antagonists to alter brain NO pathways to assess whether high plasma arginine levels in mice lacking PEPT1 indeed prevent proper leptin signalling for an adaptive increase in food intake on protein-rich diets.

In summary, we have demonstrated that *Pept1^−/−^* animals on a high-protein diet show a severe reduction in food intake compared to wildtype animals that lasts for 5 to 6 days. Thereafter a regain in food intake is observed but despite almost identical caloric intake rates like the control animals, *Pept1^−/−^* mice do not show a weight gain. This is probably a consequence of a yet unexplained reduction in energy assimilation with an increased fecal energy loss most pronounced on a high-protein diet. As a striking finding we observed significantly elevated plasma arginine levels in *Pept1^−/−^* mice on both, control and HP diet and a markedly reduced plasma leptin level only on the HP diet. Previous studies suggested a cross-talk of leptin with the arginine-dependent NO system in brain as part of the hypothalamic control loops that affect food intake. Based on our data we hypothesize that during the food intake reduction phase, the orexigenic action of low leptin levels is blunted in PEPT1-deficient animals in association with the constitutively increased plasma and possibly also brain arginine levels. Further studies in *Pept1^−/−^* mice with high-protein diets and extra injections of leptin or administration of NOS antagonists could help to identify the mechanims underlying these genotype and diet-specific effects described here.

## Materials and Methods

### Animals

Mice lacking PEPT1 were created by targeted disruption of the *Pept1* gene and obtained from Deltagen (San Mateo, California, USA) [25]. Animals were backcrossed for 10 generations to C57BL/6J background and maintained at 22±2°C and a 12∶12 h light/dark cycle. All procedures were conducted according to the German guidelines for animal care and approved by the state of Bavaria (Regierung von Oberbayern) ethics committee (Reference number: 55.2-1-54-2531-140-08).

### Study design of feeding trials for 5 or 18 days

For the feeding studies *Pept1^+/+^* and *Pept1^−/−^* animals (n = 10, per diet and genotype) received semi-synthetic purified diets with low (8% of energy; Ssniff E15202, Ssniff, Germany), medium (21% of energy; Ssniff E15000) or high (45% of energy; Ssniff E15209) protein content. Protein was isoenergetically exchanged for starch. Mice were kept individually to allow recording of food intake and water consumption. Throughout the study, mice had access to tap water and food *ad libitum*. To allow for adaptation, animals received the medium protein diet for 3 days. In the following 5 days, mice had free access to either low-protein (LP), medium = control (C) or high-protein (HP) diet. Body weight, food and water consumption were monitored daily between 8:00 and 9:00 a.m. Feces was collected daily. On the last day, blood was collected into Li-Heparin coated tubes (Sarstedt, Nümbrecht, Germany) by puncturing the retro-orbital sinus under isoflurane anaesthesia. In the second feeding trial, lasting 18 days (n = 3), only the C and HP diet were given and metabolic parameters were measured daily. Every 5 days, fecal samples were collected and on day 18 plasma and tissue samples were taken.

### Plasma amino acid analysis

By liquid chromatography-tandem mass spectrometry (LC-MS/MS) (3200QTRAP LC/MS/MS, Applied Biosystems, USA) with iTRAQ labeling for quantification, amino acids and derivatives in plasma using the AA45/32 Kit, according to the manufacturer's instructions (Applied Biosystems, USA), were determined. The data obtained were analyzed using the Analyst® 1.5 Software.

### Analysis of portal blood for labeled amino acids derived from an oral ^15^N-protein administration

Analysis of portal blood and tissue samples from jejunum, duodenum and ileum collected from *Pept1^+/+^* and *Pept1^−/−^* mice after administration of a ^15^N-yeast extract to mice via intragastric gavage have been described previously [26]. Briefly, mice (n = 6, per group and genotype) received 8.83 mg ^15^N-labeled yeast protein by gavage. After 15 and 30 min blood was collected from portal vein and plasma samples were analyzed for labeled and non-labeled amino acids via LC-MS/MS.

### Measurement of AST, ALT and GDH activities and urea

In liver samples of mice receiving the C or the HP diet, enzyme activities of aspartate aminotransferase (AST), alanine aminotransferase (ALT) and glutamate dehydrogenase (GDH) were analyzed. Samples were homogenized with 0.9% NaCl and centrifuged (15 min, 14000 *g*). Supernatant was diluted 1∶100 and enzyme activities were determined with the GPT (ALT) liquidUVTest #12012 and GOT (AST) liquiUV Test #12011 (both Human Gesellschaft für Biochemica und Diagnostica, Wiesbaden, Germany) and GDH FS #G82100 (Rolf Greiner BioChemica GmbH, Flacht, Germany) according to the manufacturer's instructions. Urea levels were analyzed from supernatant without dilution with the Urea Liquicolor Test #10505 (Human Gesellschaft für Biochemica und Diagnostica, Wiesbaden, Germany) according to the manufacturer's instructions.

### Clinical chemistry of plasma samples

A multiple analyte panel by the Piccolo xpress™ Chemistry analyzer (Abaxis, California, USA) was used for quantitative determination of albumin, alkaline phosphatase (ALP), amylase, aspartate aminotransferase (AST), calcium, creatinine, gamma glutamyltransferase (GGT), glucose, total bilirubin, total protein, blood urea nitrogen (BUN), and uric acid in plasma samples.

### Hormone levels in plasma

In plasma of *Pept1^+/+^* and *Pept1^−/−^* animals (n = 10), basal hormone levels in fed and fasted state (fasting period: 6 h) on C diet and after feeding the HP diet for 24 h were determined. Samples were collected by puncturing the retro-orbital sinus under isoflurane anaesthesia. Blood was collected in either Li-Heparin coated tubes for insulin and leptin measurements, or in EDTA-coated tubes (both Sarstedt, Nümbrecht, Germany) for ghrelin determination. For ghrelin measurement, Pefabloc^®^ SC (Sigma-Aldrich, Steinheim, Germany) was added for a final concentration of 1 mg/ml. Insulin levels were determined using the Ultra Sensitive Mouse Insulin ELISA Kit and leptin levels using the Mouse Leptin ELISA Kit (both Crystal Chem Inc., Illinois, USA). Ghrelin levels were assayed by the Rat/Mouse Ghrelin (active) ELISA Kit (Millipore GmbH, Schwalbach, Germany).

### Bomb calorimetry of feces samples

Gross energy content of feces was determined using a bomb calorimeter (Parr 6300 Calorimeter, Parr Instrument Co., Illinois, USA). Due to the low amount of feces in the feeding studies, dried feces samples were pooled (per groups) and grinded with a pebble mill (TissueLyser, Qiagen, Hilden, Germany), pressed to a pellet (∼1 g of feces) and analyzed by a bomb calorimeter.

### Statistical analysis

Statistical analysis was performed using R 2.8 (R Foundation of Statistical Computing [58]) and GraphPad Prism 4.01 (GraphPad Software, California, USA). One-way or two-way ANOVA and Tukey-test or unpaired Students *t*-test were used to test for statistical significance. Data are presented as mean±SD unless stated otherwise.

## Supporting Information

Figure S1 **Amino acid appearance in plasma after administration of a low dose of ^15^N-labeled protein.** Analysis of ^14^N and ^15^N labeled amino acids from plasma of portal vein 15 (A) and 30 min (B) after administration of ^15^N-labeled protein (8.83 mg) by gavage in 12h-fasted *Pept1^+/+^* (wt) and *Pept1* knockout (ko) animals (n = 5 animals per timepoint and genotype). Cit, citrulline; Sum, sum of all measured amino acids. (•) indicate outlier. Data are presented as mean ± SD. *P<0.05, ^$^P<0.01, ^§^P<0.001.
(TIF)

Table S1 **Plasma amino acid concentrations of male** ***Pept1^+/+^*** **and** ***Pept1^−/−^*** **animals after 5 days on control or high-protein diet.** By LC-MS/MS plasma amino acid concentrations of *Pept1^+/+^* and *Pept1^−/−^* animals on control (21% energy from protein) or high-protein (45% energy from protein) diet for 5 days were analyzed (n = 10). Data shows all analyzed amino acids plus sum of all amino acids.
(DOC)

Table S2 **Plasma amino acid concentrations of male** ***Pept1^+/+^*** **and** ***Pept1^−/−^*** **animals on control or high-protein diet for 18 days.** After feeding a control (21% energy from protein) or high-protein (45% energy from protein) diet for 18 days plasma amino acid concentrations of *Pept1^+/+^* and *Pept1^−/−^*animals were analyzed by LC-MS/MS (n = 3). Data shows all analyzed amino acids plus sum of all amino acids.
(DOC)

Table S3 **Concentrations of liver amino acids and derivatives of Pept1+/+ and Pept1−/− animals on control or high-protein diet after 18 days of feeding.** By LC-MS/MS liver amino acid concentrations of Pept1+/+ and Pept1−/− animals on control (21% energy from protein) or high-protein (45% energy from protein) diet were analyzed. Data shows all analyzed amino acids plus sum of all amino acids. Data are presented as mean±SD. (n = 3).
(DOC)
