# Supplementary material for: The Intestinal Peptide Transporter PEPT1 Is Involved in Food Intake Regulation in Mice Fed a High-Protein Diet
Source: PLoS One. 2011 Oct 21;6(10):e26407. doi: 10.1371/journal.pone.0026407 (PMC3198773; doi:10.1371/journal.pone.0026407)
Supplement: Table S1 — Plasma amino acid concentrations of male Pept1+/+ and Pept1−/− animals after 5 days on control or high-protein diet. By LC-MS/MS plasma amino acid concentrations of Pept1+/+ and Pept1−/− animals on control (21% energy from protein) or high-protein (45% energy from protein) diet for 5 days were analyzed (n = 10). Data shows all analyzed amino acids plus sum of all amino acids. (DOC) [file pone.0026407.s002.doc]

**Table S1: Plasma amino acid concentrations of male *Pept1^+/+^* and *Pept1^-/-^*** **animals after 5 days on control or high-protein diet.**

By LC-MS/MS plasma amino acid concentrations of *Pept1^+/+^* and *Pept1^-/-^* animals on control (21% energy from protein) or high-protein (45% energy from protein) diet for 5 days were analyzed (n=10). Data shows all analyzed amino acids plus sum of all amino acids.

| **Amino acid (µmol/l)** | **Control** | | **High-protein** | |
| --- | --- | --- | --- | --- |
|  | ***Pept1*^+/+^** | ***Pept1*^-/-^** | ***Pept1*^+/+^** | ***Pept1*^-/-^** |
| **Alanine** | 478.12 ± 61.28 | 481.56 ± 85.20 | 489.60 ± 101.49 | 441.00 ± 200.03 |
| **Alpha-aminoadipic acid** | 9.27 ± 2.04 | 10.81 ± 2.32 | 8.04 ± 1.82 | 9.97 ± 4.07 |
| **Arginine** | 44.77 ± 22.83^b,c^ | 90.01 ± 29.77^b^ | 53.08 ± 19.07^b^ | 96.88 ± 42.58^b,c^ |
| **Asparagine** | 60.98 ± 18.94 | 69.13 ± 32.70 | 53.07 ± 29.58 | 67.23 ± 36.94 |
| **Beta-Alanine** | 8.86 ± 4.27 | 4.80 ± 0.45 | 6.24 ± 2.09 | 7.53 ± 3.63 |
| **Citrulline** | 66.98 ± 10.49 | 80.98 ± 10.90 | 64.85 ± 18.05 | 78.97 ± 19.79 |
| **Ethanolamine** | 17.53 ± 3.70 | 17.58 ± 4.72 | 17.55 ± 2.59 | 18.31 ± 6.81 |
| **Glutamate** | 51.65 ± 12.20 | 59.34 ± 16.95 | 53.41 ± 18.09 | 44.63 ± 14.05 |
| **Glutamine** | 558.89 ± 101.88 | 529.22 ± 121.78 | 607.80 ± 138.37 | 668.60 ± 148.07 |
| **Glycine** | 204.46 ± 35.46 | 236.33 ± 124.60 | 184.60 ± 42.96 | 171.40 ± 55.35 |
| **Histidine** | 68.60 ± 13.73 | 70.72 ± 3.82 | 67.61 ± 13.83 | 80.27 ± 18.10 |
| **Hydroxyproline** | 12.46 ± 2.82 | 15.77 ± 4.31^c,d^ | 9.57 ± 1.65^c^ | 8.71 ± 4.90^d^ |
| **Isoleucine** | 154.07 ± 53.29 | 153.33 ± 32.65 | 204.70 ± 57.70 | 179.30 ± 60.76 |
| **Leucine** | 234.63 ± 81.49 | 216.11 ± 48.76 | 308.50 ± 97.64 | 288.10 ± 128.86 |
| **Lysine** | 367.83 ± 84.68 | 424.44 ± 125.50^c^ | 265.10 ± 44.01^c^ | 399.40 ± 178.29 |
| **Methionine** | 84.93 ± 55.05 | 92.12 ± 56.43 | 95.27 ± 78.26 | 80.79 ± 46.86 |
| **Ornithine** | 119.41 ± 43.40 | 101.17 ± 24.86 | 94.72 ± 26.63 | 96.26 ± 26.37 |
| **Phenylalanine** | 76.48 ± 16.27 | 76.41 ± 19.24 | 79.25 ± 14.50 | 91.66 ± 27.46 |
| **Proline** | 151.21 ± 49.46^b^ | 280.33 ± 59.60^b,c^ | 157.10 ± 46.65^c^ | 222.30 ± 146.55 |
| **Serine** | 139.88 ± 29.80 | 149.22 ± 41.95 | 124.38 ± 43.93 | 142.05 ± 54.97 |
| **Taurine** | 359.89 ± 91.45 | 377.22 ± 201.55 | 313.60 ± 90.90 | 383.40 ± 195.83 |
| **Threonine** | 232.06 ± 53.12 | 234.11 ± 43.84 | 200.80 ± 34.40 | 198.40 ± 80.66 |
| **Tryptophan** | 89.93 ± 10.97 | 81.06 ± 16.24 | 76.82 ± 13.16 | 72.88 ± 30.87 |
| **Tyrosine** | 147.48 ± 44.97 | 126.10 ± 30.04 | 126.88 ± 48.02 | 129.50 ± 95.61 |
| **Valine** | 426.22 ± 104.29^c^ | 408.00 ± 63.01^d^ | 612.40 ± 152.04^c,d^ | 480.10 ± 142.17 |
| **1-M-Histidine** | 2.82 ± 0.15 | 3.29 ± 1.10 | 2.58 ± 0.50 | 3.66 ± 0.65 |
| **3-M-Histidine** | 2.62 ± 0.56 | 2.90 ± 0.64 | 2.01 ± 0.46^b^ | 3.49 ± 0.69^b^ |
| **2-aminobutyric acid** | 9.17 ± 2.10 | 9.26 ± 2.54 | 12.86 ± 4.61 | 12.09 ± 5.52 |
| **Sum** | 4182.83 ± 618.42 | 4403.21 ± 588.29 | 4293.95 ± 763.83 | 4478.38 ± 1123.11 |

All data are presented as mean±SD

*P-value* obtained by two-factor ANOVA analysis

^b^*P<*0.05 obtained by post-hoc analysis (Tukey) when comparing between genotypes in animals consuming the same diet

^c, d^*P<*0.05 obtained by post-hoc analysis (Tukey) when comparing between diets, independently of genotype
