# Supplementary material for: The Intestinal Peptide Transporter PEPT1 Is Involved in Food Intake Regulation in Mice Fed a High-Protein Diet
Source: PLoS One. 2011 Oct 21;6(10):e26407. doi: 10.1371/journal.pone.0026407 (PMC3198773; doi:10.1371/journal.pone.0026407)
Supplement: Table S2 — Plasma amino acid concentrations of male Pept1+/+ and Pept1−/− animals on control or high-protein diet for 18 days. After feeding a control (21% energy from protein) or high-protein (45% energy from protein) diet for 18 days plasma amino acid concentrations of Pept1+/+ and Pept1−/−animals were analyzed by LC-MS/MS (n = 3). Data shows all analyzed amino acids plus sum of all amino acids. (DOC) [file pone.0026407.s003.doc]

**Table S2: Plasma amino acid concentrations of male *Pept1^+/+^* and *Pept1*^-/-^ animals on control or high-protein diet for 18 days.**

After feeding a control (21% energy from protein) or high-protein (45% energy from protein) diet for 18 days plasma amino acid concentrations of *Pept1^+/+^* and *Pept1^-/-^* animals were analyzed by LC-MS/MS (n=3). Data shows all analyzed amino acids plus sum of all amino acids.

| **Amino acid (µmol/l)** | **Control** | | **High protein** | |
| --- | --- | --- | --- | --- |
|  | ***Pept1*^+/+^** | ***Pept1*^-/-^** | ***Pept1*^+/+^** | ***Pept1*^-/-^** |
| **Alanine** | 557.67 ± 166.64 | 592.67 ± 225.31 | 521.67 ± 103.32 | 449.67 ± 52.56 |
| **Alpha-aminoadipic acid** | 10.81 ± 2.77 | 9.90 ± 5.02 | 7.79 ± 0.74 | 11.38 ± 1.44 |
| **Arginine** | 29.93 ± 16.07^b,c^ | 76.80 ± 18.49^b^ | 56.30 ± 12.56 | 66.97 ± 3.72^c^ |
| **Asparagine** | 77.37 ± 25.89 | 127.17 ± 58.77 | 62.50 ± 14.19 | 71.23 ± 12.34 |
| **Aspartate** | 4.80 ± 0.66 | 8.58 ± 7.12 | 7.74 ± 0.64 | 6.66 ± 2.68 |
| **Citrulline** | 61.90 ± 7.83 | 83.67 ± 9.01^c^ | 61.10 ± 18.40 | 52.27 ± 2.51^c^ |
| **Ethanolamine** | 20.40 ± 2.95 | 22.47 ± 5.59 | 16.00 ± 3.56 | 16.33 ± 3.03 |
| **Gamma-aminobutyric acid** | 1.73 ± 0.33 | 1.52 ± 0.32 | 1.55 ± 0.17 | 1.46 ± 0.07 |
| **Glutamate** | 79.43 ± 17.07 | 94.97 ± 53.00 | 78.33 ± 6.27 | 68.10 ± 16.46 |
| **Glutamine** | 751.33 ± 21.94 | 760.33 ± 143.27 | 695.33 ± 25.50 | 628.33 ± 37.17 |
| **Glycine** | 283.33 ± 40.15 | 285.00 ± 128.99 | 209.33 ± 23.18 | 215.00 ± 16.52 |
| **Histidine** | 71.87 ± 5.65 | 97.93 ± 18.26 | 69.30 ± 9.72 | 67.70 ± 9.91 |
| **Homocysteine** | 3.19 ± 0.19 | 2.94 ± 0.12 | 3.05 ± 0.16 | 3.06 ± 0.14 |
| **Hydroxyproline** | 13.77 ± 2.20^c^ | 10.25 ± 2.09 | 8.86 ± 0.95^c^ | 12.43 ± 1.19 |
| **Isoleucine** | 143.00 ± 34.39 | 138.33 ± 7.23 | 264.67 ± 87.09 | 167.33 ± 33.25 |
| **Leucine** | 213.33 ± 51.25^c^ | 197.33 ± 14.19^d^ | 413.67 ± 124.84^c,d^ | 264.67 ± 45.54 |
| **Lysine** | 326.33 ± 90.14 | 304.67 ± 89.63 | 252.00 ± 65.21 | 249.67 ± 40.67 |
| **Methionine** | 49.80 ± 8.66 | 63.03 ± 18.74 | 50.97 ± 18.57 | 31.90 ± 10.04 |
| **Ornithine** | 134.03 ± 32.99 | 86.90 ± 11.76 | 106.27 ± 30.64 | 81.40 ± 17.87 |
| **Phenylalanine** | 83.50 ± 17.91 | 100.90 ± 18.37 | 97.50 ± 14.53 | 77.47 ± 10.25 |
| **Phosphoethanolamine** | 0.36 ± 0.06 | 0.39 ± 0.05 | 0.37 ± 0.11 | 0.56 ± 0.33 |
| **Proline** | 111.93 ± 49.85 | 219.00 ± 88.15 | 161.63 ± 84.95 | 160.33 ± 50.52 |
| **Serine** | 163.00 ± 46.87 | 187.33 ± 78.87 | 127.67 ± 21.83 | 130.67 ± 30.75 |
| **Taurine** | 390.67 ± 83.99 | 315.33 ± 47.61 | 368.67 ± 116.14 | 387.33 ± 34.53 |
| **Threonine** | 221.00 ± 77.31 | 179.33 ± 42.85 | 205.33 ± 65.06 | 184.67 ± 40.28 |
| **Tryptophan** | 104.87 ± 23.53 | 95.27 ± 11.75 | 89.13 ± 17.69 | 72.50 ± 6.29 |
| **Tyrosine** | 99.83 ± 39.42 | 153.00 ± 31.61 | 129.03 ± 38.65 | 73.77 ± 18.58 |
| **Valine** | 341.33 ± 77.36^c^ | 379.67 ± 37.90 | 682.33 ± 213.01^c^ | 417.00 ± 96.89 |
| **1-M-Histidine** | 4.25 ± 0.82b | 2.71 ± 0.23^b^ | 3.18 ± 0.08 | 3.75 ± 0.38 |
| **3-M-Histidine** | 3.31 ± 0.60^b^ | 1.94 ± 0.21^b,c^ | 2.30 ± 0.45 | 3.33 ± 0.34^c^ |
| **2-aminobutyric acid** | 7.42 ± 1.54 | 6.49 ± 0.47 | 11.73 ± 3.72 | 11.93 ± 3.46 |
| **Sum** | 4365.51 ± 891.54 | 4605.83 ± 721.49 | 4765.30 ± 1019.24 | 3988.85 ± 426.01 |

All data are presented as mean±SD

*P-value* obtained by two-factor ANOVA analysis

^b^*P<*0.05 obtained by post-hoc analysis (Tukey) when comparing between genotypes genotypes in animals consuming the same diet

^c, d^*P<*0.05 obtained by post-hoc analysis (Tukey) when comparing between diets, independently of genotype
