# Supplementary material for: The Intestinal Peptide Transporter PEPT1 Is Involved in Food Intake Regulation in Mice Fed a High-Protein Diet
Source: PLoS One. 2011 Oct 21;6(10):e26407. doi: 10.1371/journal.pone.0026407 (PMC3198773; doi:10.1371/journal.pone.0026407)
Supplement: Table S3 — Concentrations of liver amino acids and derivatives of Pept1+/+ and Pept1−/− animals on control or high-protein diet after 18 days of feeding. By LC-MS/MS liver amino acid concentrations of Pept1+/+ and Pept1−/− animals on control (21% energy from protein) or high-protein (45% energy from protein) diet were analyzed. Data shows all analyzed amino acids plus sum of all amino acids. Data are presented as mean±SD. (n = 3). (DOC) [file pone.0026407.s004.doc]

**Table S3: Concentrations of liver amino acids and derivatives of *Pept1^+/+^* and *Pept1^-/-^*****animals on control or high-protein diet after 18 days of feeding.**

By LC-MS/MS liver amino acid concentrations of *Pept1^+/+^* and *Pept1^-/-^* animals on control (21% energy from protein) or high-protein (45% energy from protein) diet were analyzed. Data shows all analyzed amino acids plus sum of all amino acids. Data are presented as mean±SD. (n=3).

| **Amino acid (nmol/mg protein)** | **Control** | | **High-protein** | |
| --- | --- | --- | --- | --- |
|  | ***Pept1*^+/+^** | ***Pept1*^-/-^** | ***Pept1*^+/+^** | ***Pept1*^-/-^** |
| **Alanine** | 83.90 ± 10.28 | 92.21 ± 15.87 | 96.58 ± 28.53 | 84.43 ± 17.13 |
| **Alpha-aminoadipic acid** | 0.84 ± 0.61 | 1.23 ± 0.56 | 0.88 ± 0.70 | 0.96 ± 0.67 |
| **Alpha-aminoisobutyric acid** | 0.19 ± 0.10 | 0.25 ± 0.06 | 0.30 ± 0.12 | 0.25 ± 0.03 |
| **Asparagine** | 2.70 ± 0.45 | 3.10 ± 0.98 | 3.69 ± 0.43 | 4.82 ± 1.73 |
| **Aspartate** | 4.25 ± 1.97 | 5.74 ± 3.22 | 2.78 ± 0.26 | 7.25 ± 4.18 |
| **Citrulline** | 1.24 ± 0.28^b^ | 3.64 ± 1.78^b,c,d^ | 0.41 ± 0.12^c^ | 0.39 ± 0.13^d^ |
| **Ethanolamine** | 0.26 ± 0.05 | 0.42 ± 0.12 | 1.71 ± 0.91 | 1.51 ± 0.65 |
| **Gamma-aminobutyric acid** | 0.28 ± 0.01 | 0.32 ± 0.10 | 0.44 ± 0.07 | 0.56 ± 0.22 |
| **Glutamate** | 20.07 ± 1.52 | 35.57 ± 18.04 | 19.56 ± 6.12 | 25.92 ± 10.23 |
| **Glutamine** | 78.80 ± 6.35^b,c^ | 44.98 ± 3.48^b^ | 54.13 ± 5.42^c^ | 62.81 ± 12.42 |
| **Glycine** | 46.90 ± 3.02 | 53.05 ± 27.75 | 49.59 ± 2.59 | 55.48 ± 11.02 |
| **Histidine** | 8.93 ± 1.37 | 10.30 ± 2.07 | 7.39 ± 1.27 | 9.32 ± 3.12 |
| **Isoleucine** | 4.28 ± 0.62 | 4.89 ± 1.75 | 6.51 ± 2.34 | 6.12 ± 2.64 |
| **Leucine** | 6.98 ± 1.01 | 8.17 ± 2.82 | 11.09 ± 2.62 | 11.71 ± 4.61 |
| **Lysine** | 8.18 ± 1.06 | 9.61 ± 2.15 | 10.46 ± 2.21 | 13.64 ± 5.92 |
| **Methionine** | 3.21 ± 1.36 | 4.89 ± 0.69^c,d^ | 1.59 ± 0.19^c^ | 1.74 ± 0.29^d^ |
| **Ornithine** | 2.36 ± 0.37 | 2.98 ± 1.40 | 5.18 ± 1.25 | 7.10 ± 4.33 |
| **Phenylalanine** | 2.29 ± 0.31 | 2.47 ± 0.55 | 2.69 ± 0.39 | 3.42 ± 1.37 |
| **Phosphoethanolamine** | 3.47 ± 0.57 | 7.09 ± 3.38 | 3.48 ± 0.79 | 5.04 ± 0.53 |
| **Proline** | 5.22 ± 1.63 | 7.78 ± 2.54 | 6.39 ± 3.67 | 7.63 ± 4.24 |
| **Sarcosine** | 0.88 ± 0.44 | 1.11 ± 0.34 | 0.65 ± 0.55 | 0.59 ± 0.47 |
| **Serine** | 3.83 ± 0.05^c^ | 2.83 ± 0.60^d^ | 6.28 ± 0.67 | 10.11 ± 4.16^c,d^ |
| **Taurine** | 236.97 ± 20.45 | 269.57 ± 70.91 | 186.68 ± 28.41 | 191.41 ± 32.30 |
| **Threonine** | 4.89 ± 1.00 | 4.35 ± 1.20 | 4.87 ± 0.55 | 8.82 ± 4.38 |
| **Tryptophan** | 0.76 ± 0.17 | 0.95 ± 0.26 | 1.06 ± 0.25 | 0.96 ± 0.25 |
| **Tyrosine** | 2.58 ± 0.66 | 3.73 ± 1.18 | 4.78 ± 1.15 | 5.11 ± 2.05 |
| **Valine** | 8.84 ± 1.15 | 13.71 ± 5.17 | 14.33 ± 6.20 | 13.97 ± 6.95 |
| **2-Aminobutyric acid** | 0.75 ± 0.14 | 2.22 ± 1.48 | 2.16 ± 1.03 | 2.21 ± 1.04 |
| **Sum** | 543.83 ± 19.72 | 597.16 ± 147.47 | 505.66 ± 85.14 | 543.27 ± 115.73 |

All data are presented as mean±SD

*P-value* obtained by two-factor ANOVA analysis

^b^*P<*0.05 obtained by post-hoc analysis (Tukey) when comparing between genotypes genotypes in animals consuming the same diet

^c, d^*P<*0.05 obtained by post-hoc analysis (Tukey) when comparing between diets, independently of genotype
